# Comparison of Three Clinical Trial Treatments for Autism Spectrum Disorder Through Multivariate Analysis of Changes in Metabolic Profiles and Adaptive Behavior

**DOI:** 10.3389/fncel.2018.00503

**Published:** 2018-12-19

**Authors:** Troy Vargason, Uwe Kruger, Emily Roth, Leanna M. Delhey, Marie Tippett, Shannon Rose, Sirish C. Bennuri, John C. Slattery, Stepan Melnyk, S. Jill James, Richard E. Frye, Juergen Hahn

**Affiliations:** ^1^Department of Biomedical Engineering, Rensselaer Polytechnic Institute, Troy, NY, United States; ^2^Center for Biotechnology and Interdisciplinary Studies, Rensselaer Polytechnic Institute, Troy, NY, United States; ^3^Arkansas Children’s Research Institute, Little Rock, AR, United States; ^4^Department of Epidemiology, Fay W. Boozman College of Public Health, University of Arkansas for Medical Sciences, Little Rock, AR, United States; ^5^Department of Pediatrics, University of Arkansas for Medical Sciences, Little Rock, AR, United States; ^6^BioROSA Technologies, Inc., San Francisco, CA, United States; ^7^Department of Child Health, University of Arizona College of Medicine, Phoenix, AZ, United States; ^8^Phoenix Children’s Hospital, Phoenix, AZ, United States; ^9^Department of Chemical and Biological Engineering, Rensselaer Polytechnic Institute, Troy, NY, United States

**Keywords:** autism spectrum disorder, multivariate analysis, one-carbon metabolism, transsulfuration, adaptive behavior, methylcobalamin, tetrahydrobiopterin, folinic acid

## Abstract

Several studies associate autism spectrum disorder (ASD) pathophysiology with metabolic abnormalities related to DNA methylation and intracellular redox homeostasis. In this regard, three completed clinical trials are reexamined in this work: treatment with (i) methylcobalamin (MeCbl) in combination with low-dose folinic acid (LDFA), (ii) tetrahydrobiopterin, and (iii) high-dose folinic acid (HDFA) for counteracting abnormalities in the folate-dependent one-carbon metabolism (FOCM) and transsulfuration (TS) pathways and also for improving ASD-related symptoms and behaviors. Although effects of treatment on individual metabolites and behavioral measures have previously been investigated, this study is the first to consider the effect of interventions on a set of metabolites of the FOCM/TS pathways and to correlate FOCM/TS metabolic changes with behavioral improvements across several studies. To do so, this work uses data from one case–control study and the three clinical trials to develop multivariate models for considering these aspects of treatment. Fisher discriminant analysis (FDA) is first used to establish a model for distinguishing individuals with ASD from typically developing (TD) controls, which is subsequently evaluated on the three treatment data sets, along with one data set for a placebo, to characterize the shift of FOCM/TS metabolism toward that of the TD population. Treatment with MeCbl plus LDFA and, separately, treatment with tetrahydrobiopterin significantly shifted the metabolites toward the values of the control group. Contrary to this, treatment with HDFA had a lesser, though still noticeable, effect whilst the placebo group showed marginal, but not insignificant, variations in metabolites. A second analysis is then performed with non-linear kernel partial least squares (KPLS) regression to predict changes in adaptive behavior, quantified by the Vineland Adaptive Behavior Composite, from changes in FOCM/TS biochemical measurements provided by treatment. Incorporating the 74 samples receiving any treatment, including placebo, into the regression analysis yields an *R*^2^ of 0.471 after cross-validation when using changes in six metabolic measurements as predictors. These results are suggestive of an ability to effectively improve pathway-wide FOCM/TS metabolic and behavioral abnormalities in ASD with clinical treatment.

## Introduction

Deficits in communication and behavior are the defining characteristics of autism spectrum disorder (ASD) (American Psychiatric Association, 2013), a neurodevelopmental disorder estimated by the Centers for Disease Control and Prevention to affect one out of 59 children in the United States (Baio et al., 2018). The national economic burden of ASD in 2015 was calculated to be $268 billion, similar to the costs of diabetes and attention deficit hyperactivity disorder (Leigh and Du, 2015). ASD is a highly heterogeneous disorder in terms of how it presents itself in each individual, with as many as 95% of diagnosed children also affected by at least one co-occurring condition (Soke et al., 2018) and regressive forms of the disorder not being uncommon (Ozonoff et al., 2018). Despite the large body of research investigating the etiology of ASD, there is relatively limited understanding of the pathophysiology of the disorder aside from complex interactions between genetic and environmental contributors being involved (Gaugler et al., 2014).

As a result of the heterogeneity and lack of biological understanding of ASD, the current standards for diagnosis are clinical evaluations of patient behavior, which while comprehensive do not offer the objective assessment of ASD status that a biomarker can offer. A consequence of this gap in knowledge is initial ASD diagnoses being made at a median age of 4 years (Baio et al., 2018) even though stable diagnoses have been shown to be possible at 2 years of age in a large percentage of children (Ozonoff et al., 2015). Given that earlier behavioral intervention typically leads to milder ASD-related symptoms and improved development of social and behavioral skills later in life (Fein et al., 2017), it is of great interest to achieve improved methods of ASD screening. Identification of biological markers for diagnosing ASD or assessing ASD risk status would thus represent a significant step toward improving long-term outcomes in individuals with ASD.

Potential biomarkers for ASD diagnosis may involve the folate-dependent one-carbon metabolism (FOCM) and transsulfuration (TS) pathways as these pathways have been linked to metabolic abnormalities in ASD in several studies. Case–control studies show that markers of DNA methylation and intracellular redox status are significantly different in individuals with ASD compared to typically developing (TD) peers (James et al., 2006; Melnyk et al., 2012), suggesting perturbations both in the epigenetic control of gene expression and in the control of intracellular oxidative stress. Subsequent studies have found a strong ability to classify individuals as having ASD or being TD, as well as predict adaptive behavior, based on these measurements (Howsmon et al., 2017, 2018; Li et al., 2018). Development of a mathematical model of these pathways with parameters estimated from clinical data has also pointed to several metabolic reactions that may be disrupted in individuals with ASD (Vargason et al., 2017b).

Aside from investigating FOCM/TS metabolites for diagnostic purposes, correcting activity in the FOCM and TS pathways may affect underlying biological processes that contribute to ASD pathophysiology, thus making metabolic abnormalities in these pathways promising targets for clinical treatment (Frye and Rossignol, 2016). Furthermore, it has been suggested that early detection of metabolic dysfunction to determine ASD risk and allowing for proactive treatment strategies could potentially lead to practical intervention plans for at least a subset of those at risk for ASD (Slattery et al., 2016). Since the aim of treatment is not just to correct metabolic abnormalities but also to alleviate the primary behavioral symptoms of ASD, it would be of great value to determine treatment targets where improvements in metabolic activity give rise to amelioration of observed behavior. Previous studies by the authors have investigated the effects of treatment with methylcobalamin (MeCbl) in combination with low-dose folinic acid (LDFA) (James et al., 2009), tetrahydrobiopterin (BH_4_) (Frye et al., 2013a), and high-dose folinic acid (HDFA) (Frye et al., 2016b) for improving metabolic and behavioral outcomes in individuals with ASD (Delhey et al., 2018). The growing body of literature describing the efficacy of these treatments suggests unique mechanisms by which each acts upon metabolic pathways that may be dysfunctional in ASD.

Methylcobalamin, one treatment option for ASD that has been explored, is a cofactor for the methionine synthase enzyme that contributes to the process of DNA methylation. Levels of methionine synthase messenger RNA in the frontal cortex typically decrease with age, but this decrease has been found to occur more quickly in ASD even though actual levels of the enzyme do not appear to be affected significantly (Muratore et al., 2013). Concentrations of MeCbl in the frontal cortex of children with ASD have been measured to be three times lower than those in TD individuals, with an associated threefold decrease in methionine synthase activity also measured (Zhang et al., 2016). It has been suggested that cobalamin transporter polymorphisms and mutations (James et al., 2006; Nashabat et al., 2017) may contribute to the development of ASD. Open-label (James et al., 2009; Frye et al., 2013b) and double-blind placebo-controlled (Bertoglio et al., 2010; Hendren et al., 2016) studies of MeCbl treatment have observed improvement in metabolism and ASD-related symptoms in children with the disorder.

Another studied treatment for ASD involves BH_4_, which has diverse roles in monoamine neurotransmitter production, phenylalanine breakdown, and nitric oxide synthesis (Frye et al., 2010). Reduced cerebrospinal fluid levels of BH_4_ have been reported in children with ASD, with one study reporting these levels to be only 42% of those found in TD children (Tani et al., 1994) and a small open-label trial of BH_4_ requiring deficient levels as an inclusion criterion (Fernell et al., 1997). Analysis of genes related to BH_4_ pathways has suggested that the synthesis of BH_4_ may be impaired in individuals with ASD (Schnetz-Boutaud et al., 2009). One double-blind placebo-controlled study with BH_4_ observed increases in social interaction after 6 months of treatment (Danfors et al., 2005), while a more recent trial described significant improvements in ASD-related mannerisms, hyperactivity, inappropriate speech, and social awareness (Klaiman et al., 2013). Although it is unclear which underlying biological mechanisms are targeted by BH_4_ treatment, its therapeutic effect may derive from its correction of oxidative stress and overall folate metabolism in the central nervous system (Frye, 2014).

Folinic acid is also a potential treatment for ASD and is a naturally occurring form of folate, which is required for purine and pyrimidine productions, aids in the transfer of carbon during the process of amino acid synthesis, and contributes to DNA methylation processes (Wagner, 2001). Early studies of folate deficiency in the central nervous system indicated a potential connection to cases of ASD and other neurological deficits (Ramaekers et al., 2007; Moretti et al., 2008), with later studies also reporting increased levels of folate receptor autoantibodies in the blood to be correlated with the presentation of ASD-related symptoms and physiology (Frye et al., 2013c, 2016a). Additionally, higher rates of developmental deficits and ASD-like behaviors have been observed in animal models administered folate receptor antibodies during gestation and the pre-weaning period (Sequeira et al., 2016; Desai et al., 2017). The use of folate supplements during pregnancy may serve to combat these deleterious effects as it has been associated with a reduced risk of ASD in the child (Schmidt et al., 2012; Surén et al., 2013; Wang et al., 2017); this is likely due to folate’s protective effect for proper neural tube development (Blom, 2009; Schmidt et al., 2011; Frye et al., 2017). Treatment with folinic acid has also been found to correct certain abnormalities of the cerebrospinal fluid and improve ASD-related symptoms and behavior (Moretti et al., 2005; Ramaekers et al., 2007; Frye et al., 2016b).

Even though a number of studies have tested the effect of treatment on individually measured compounds or on certain behavioral measures in individuals with ASD, no study exists that investigates the effect of a treatment on combinations of metabolites of the FOCM/TS pathways and correlates pathway-wide changes to shifts in behavioral measures. This work addresses this point by using multivariate analysis on the metabolites of FOCM/TS in order to compare the effectiveness of (i) MeCbl + LDFA (James et al., 2009), (ii) BH_4_ (Frye et al., 2013a), (iii) HDFA (Frye et al., 2016b), and (iv) placebo treatment for normalizing the metabolic profiles of individual with ASD to more closely resemble those of TD individuals. Furthermore, a correlation is developed between changes in the metabolites and changes in adaptive behavior, as measured by the Vineland Adaptive Behavior Composite, that are brought about by these treatments.

## Materials and Methods

### Description of Data Sets

This study makes use of four data sets describing plasma FOCM/TS measurements from previous separately investigated and published studies. All data used here are pre-existing and de-identified. The recommendations of the respective Institutional Review Boards (IRBs) described below were followed, with study protocols also approved by the respective IRBs. Written informed consent was provided by parents of study participants and assent was given by participants themselves, when appropriate, in accordance with the Declaration of Helsinki.

#### Case–Control Data

The current study uses case–control data from the Integrated Metabolic and Genomic Endeavor (IMAGE) study at Arkansas Children’s Research Institute (Melnyk et al., 2012). The case–control group consisted of children between 3 and 10 years of age with a diagnosis of autistic disorder according to the *Diagnostic and Statistical Manual of Mental Disorders, Fourth Edition* (DSM-IV) (American Psychiatric Association, 2000), the Autism Diagnostic Observation Schedule, and/or the Childhood Autism Rating Scales (score greater than 30). TD controls were age-matched and had no indications of behavioral or neurological disorders as reported by their parents. In the ASD cohort, 85% of participants were male while 48% of the TD cohort was male. The protocol for this study was approved by the IRB at the University of Arkansas for Medical Sciences in Little Rock, AR, United States.

#### MeCbl + LDFA Treatment Data

Subcutaneously injected MeCbl (75 μg/kg, once every 3 days) in combination with oral LDFA (400 μg, twice per day) was given to children with autism in a 12-week open-label trial (James et al., 2009). Included children were aged 2–7 years and met the diagnostic criteria for autism as defined by the DSM-IV in addition to having a Childhood Autism Rating Scales score greater than 30. Boys and girls made up 82 and 18% of participants in this study, respectively. The IRB at the University of Arkansas for Medical Sciences approved the protocol for this study. This MeCbl + LDFA trial was registered at clinicaltrials.gov as NCT00692315.

#### BH_4_ Treatment Data

A 16-week open-label trial (Frye et al., 2013a) investigated the effects of orally administered BH_4_ (20 mg/kg, once per day) in children aged 2–6 years old with a previous diagnosis of ASD that was confirmed at the time of evaluation with DSM-IV criteria. Included children also needed to exhibit social or language delays and have normal concentrations of BH_4_ in their cerebrospinal fluid. Study participants were 90% males. Approval for this study was given by the IRB at the University of Texas Health Science Center at Houston, TX, United States. One note of importance is that FOCM/TS markers were measured at 8 and 16 weeks following the onset of treatment in this trial; to maintain consistency with the other trials where markers were measured after 12 weeks, the averages of the measurements taken at 8 and 16 weeks were used. This trial was registered at clinicaltrials.gov as NCT01141595.

#### HDFA Treatment Data

This study involved a double-blind placebo-controlled trial of HDFA (2 mg/kg per day up to a maximum of 50 mg daily, given orally) administered over 12 weeks to children between 3 and 14 years of age (Frye et al., 2016b). ASD diagnoses were made using the Autism Diagnostic Observation Schedule and/or Autism Diagnostic Interview – Revised, or by agreement between physician, psychologist, and speech therapist, or by a physician’s diagnosis according to the *Diagnostic and Statistical Manual of Mental Disorders, Fifth Edition* (American Psychiatric Association, 2013) with later confirmation by the investigators. All children were required to have documented language impairment. In this study, 78% of the treatment group was male while 80% of the placebo group was male. The protocol was approved by the IRB at the University of Arkansas for Medical Sciences. All data for participants receiving a placebo in the current analysis were provided by this study. The HDFA trial was registered at clinicaltrials.gov as NCT01602016.

### Key Study Variables

#### Biochemical Measurements

Concentrations and ratios of metabolites in the FOCM and TS pathways were measured in each study, with 15 measurements appearing in all four data sets. Six of these measures were associated with DNA methylation: methionine, *S*-adenosylmethionine (SAM), *S*-adenosylhomocysteine (SAH), the SAM/SAH ratio (an indicator of DNA methylation capacity), homocysteine, and adenosine. The remaining nine measures were precursors of glutathione or markers of redox metabolism: total cysteine, glutamylcysteine (Glu-Cys), cysteinylglycine (Cys-Gly), total and free reduced glutathione (tGSH and fGSH, respectively), oxidized glutathione (GSSG), the ratios of total and free reduced glutathione to oxidized glutathione (tGSH/GSSG and fGSH/GSSG, respectively; these are indicators of intracellular oxidative stress), and percent oxidized glutathione [a derived measure calculated as 2GSSG/(GSH + 2GSSG)].

#### Adaptive Behavior Assessment

The Vineland Adaptive Behavior Scales (VABS) (Sparrow et al., 2005) were used in all studies to measure adaptive behavior in the communication, daily living, and social subdomains. This work only made use of the VABS Composite score, which incorporates these subdomains to provide a single measure of adaptive behavior. Higher scores indicate better development of adaptive behavior.

#### Inclusion Criteria for the Current Study

Participants of the IMAGE study were included in the current analysis if they had a complete panel of the fifteen FOCM/TS markers of interest. Ninety-two participants with ASD and 82 TD controls met this criterion and were thus considered for further analysis. Participants of the clinical trials were included if they had complete pre- and post-treatment measurements for these fifteen markers in addition to pre- and post-treatment VABS Composite scores. Meeting these criteria were 33 participants receiving MeCbl + LDFA, eight participants receiving BH_4_, 14 participants receiving HDFA, and 19 participants receiving a placebo (74 participants with ASD in total). This information is summarized in Table 1.

**Table 1:** Participant numbers from the four data sets used in this study.

Study	ASD participants	TD participants	Inclusion criteria
IMAGE case–control	92	82	Complete panel of 15 FOCM/TS measurements
MeCbl + LDFA trial	33	0	Complete pre- and post-treatment panel of 15 FOCM/TS measurements with VABS Composite
BH_4_ trial	8	0	
HDFA trial	14 (+19 placebo)	0	
Placebo^∗^	19	0	


*^∗^All members of the placebo group analyzed in this study came from the HDFA trial.*

### Multivariate Statistical Analysis

The analytical techniques featured in this study were coded in MATLAB as part of a previous investigation (Howsmon et al., 2017) and modified for the data used in this work. All data used for model training were normalized such that each FOCM/TS marker had a mean of zero and a standard deviation of one across all training samples. Model validation samples were then normalized according to the mean/standard deviation parameters used for normalization of the training data.

#### Classification

##### Fisher discriminant analysis

Individuals of the IMAGE study (Melnyk et al., 2012) were separated into ASD and TD cohorts using Fisher discriminant analysis (FDA), a method of dimensionality reduction that maximizes the differences between several groups. FDA uses the data matrix ***X*** of size *n* × *m* as input, where *n* study participants are each defined by *m* biochemical measurements. Sample information for study participant *i* is contained in row vector ***x****_i_* (size 1 × *m*) and that participant’s value for measurement *j* is indicated by *x_i,j_*. The input matrix ***X*** can be considered as two matrices ***X***_ASD_ and ***X***_TD_ taken to represent the separate samples for the ASD and TD cohorts, respectively, with ***X***_ASD_ composed of *n*_ASD_ samples and ***X***_TD_ having *n*_TD_ samples. For the two-class problem presented here, FDA defines the between-class scatter matrix ***S****_B_* (size *m* × *m*) as follows:

$$S_{B}=n_{\mathrm{ASD}}{\left({\bar{x}}_{\mathrm{ASD}}-\bar{x}\right)}^{T}({\bar{x}}_{\mathrm{ASD}}-\bar{x})+n_{\mathrm{TD}}{\left({\bar{x}}_{\mathrm{TD}}-\bar{x}\right)}^{T}({\bar{x}}_{\mathrm{TD}}-\bar{x})$$

where ${\bar{x}}_{\mathrm{ASD}}$ denotes the mean vector of samples in ***X***_ASD_, ${\bar{x}}_{\mathrm{TD}}$ represents the mean vector among samples in ***X***_TD_, and $\bar{x}$ indicates the mean vector across all samples in ***X***. The within-class scatter matrix ***S****_W_* (size *m* × *m*) is then defined as follows:

$$S_{W}=n_{\mathrm{ASD}}\sum_{i=1}^{n_{\mathrm{ASD}}}{\left(x_{i}-{\bar{x}}_{\mathrm{ASD}}\right)}^{T}(x_{i}-{\bar{x}}_{\mathrm{ASD}})+n_{\mathrm{TD}}\sum_{i=1}^{n_{\mathrm{TD}}}{\left(x_{i}-{\bar{x}}_{\mathrm{TD}}\right)}^{T}(x_{i}-{\bar{x}}_{\mathrm{TD}})$$

where ***x****_i_* represents an individual sample from either the ASD or TD cohort. Using this information, FDA determines the *m* × 1 projection vector ***w*** that satisfies the objective function

$$\underset{w}{\max}\frac{w^{T}S_{B}w}{w^{T}S_{W}w}\to Jw=S_{W}^{-1}S_{B}w$$

where the optimal solution is given by the eigenvector of the matrix product $S_{W}^{-1}$S_B_. A final discriminant score *t_i_*, which is the projection of the *i*th data point onto the projection vector ***w***, is given by

$$t_{i}=x_{i}\cdot w=x_{i,1}w_{1}+x_{i,2}w_{2}+\cdot \cdot \cdot +x_{i,m}w_{m}.$$

Given the sets of discriminant scores ***t***_ASD_ and ***t***_TD_ for samples in the ASD and TD cohorts, respectively, the next step of the analysis is to consider the distributions of these scores and determine a rule by which individuals can be classified into each cohort.

##### Kernel density estimation

Kernel density estimation estimates the underlying probability density functions (PDFs) of discriminant scores for the ASD and TD populations using the scores from FDA as reference samples. Kernel density estimation assumes that samples not included in the estimation of a PDF will likely be near the reference samples that were used (Silverman, 1986). As part of the estimation procedure, a Gaussian kernel function is centered on each reference sample; the sum of the kernel functions associated with the samples of a particular cohort is then taken to be representative of that cohort’s total PDF. The shapes of the estimated PDFs can be adjusted by optimally determining the kernel parameter.

##### Null hypothesis for classification

The null hypothesis, *H*_0_, for classification states that a participant belongs to the TD group. With this hypothesis, the Type I (false positive) error is the probability of incorrectly classifying a TD participant as having ASD. The Type II (false negative) error is then the probability of incorrectly classifying a participant with ASD as being TD. These errors’ magnitudes are dictated by the choice of the discriminant score threshold for *H*_0_ and the amount of overlap between the PDFs for the two cohorts. In order to balance the Type I and Type II errors, the analysis presented here will place the threshold *H*_0_ at the point where the absolute difference between these errors in the fitted model is minimized.

##### FDA model evaluation of treatment data

**FIGURE 1:**
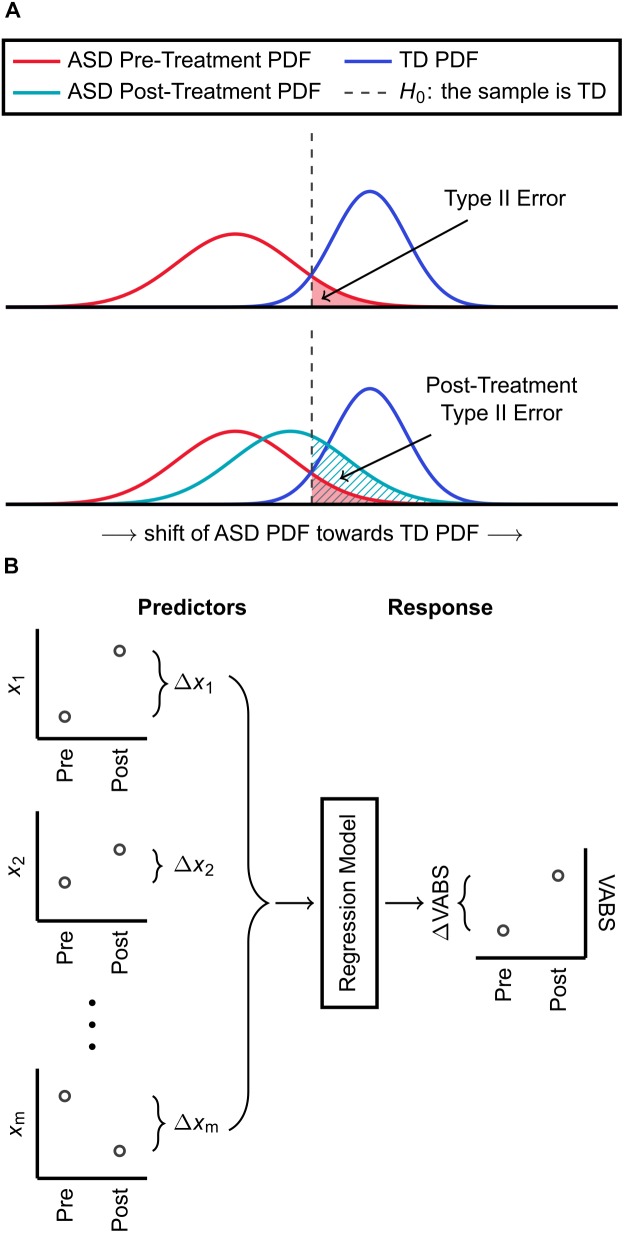
Summary of methods used for classification and regression. **(A)** An FDA model is identified for distinguishing ASD and TD cohorts based on a subset of their biochemical measurements. The estimated PDFs of discriminant scores are used to determine the threshold *H*_0_ for classification, which has an associated Type II (false negative) error. Using the same FDA model with the same subset of biochemical measurements, participants with ASD who received a treatment are also each assigned a discriminant score. The Type II error, with respect to *H*_0_, accompanying the PDF of these post-treatment scores is then calculated. An increase in Type II error due to treatment indicates that the ASD PDF shifted toward the TD PDF and that the treatment had a net corrective effect on FOCM/TS metabolism. **(B)** Pre-to-post treatment differences in *m* metabolic measurements are used as predictor variables for KPLS regression. The regression model predicts the pre-to-post treatment change in the VABS Composite score, which is the response variable, from the changes in metabolic measurements.

Using the FDA model identified from the IMAGE data and based on the same subset of FOCM/TS measurements, pre- and post-treatment discriminant scores were calculated for individuals with ASD who received the MeCbl + LDFA, BH_4_, HDFA, and placebo treatments. Pre- and post-treatment Type II errors with respect to *H*_0_, which was previously determined from model fitting involving data from the IMAGE study, were then computed for the estimated PDFs of pre- and post-treatment discriminant scores (separately for each treatment). The change in Type II error yielded by each treatment was used to quantify the abilities of these treatments to shift the metabolic profiles of individuals with ASD to be more, or less, similar to those of the TD cohort (Figure 1A). It must be emphasized that an increase in Type II error, while undesirable in traditional hypothesis testing, is considered a desirable outcome in this particular analysis as the aim, at least in theory, is to make the PDF of participants with ASD indistinguishable from the PDF of TD participants on the basis of their metabolic measurements.

##### Treatment effect sizes

The effect size for each treatment was calculated as the median pre-to-post-treatment change in discriminant score, with each participant’s pre-treatment score paired with their post-treatment score. The distribution of the effect size was obtained by bootstrap resampling (i.e., random sampling, with replacement, for a sample set equal in size to the original set) with 10,000 replications, and the 0.025 and 0.975 quantiles of the bootstrap distribution described the 95% confidence interval (CI) for the effect size (Banjanovic and Osborne, 2016).

#### Regression

Regression analysis aims to predict changes in the VABS Composite score from changes in metabolic measurements resulting from clinical treatment of individuals with ASD. For this objective, kernel partial least squares (KPLS), a non-linear extension of the partial least squares (PLS) algorithm, was used (Rosipal and Trejo, 2001). KPLS regression handles noisy and collinear data well compared to ordinary least squares and is a more appropriate choice when the number of observations is small compared to the number of variables (Höskuldsson, 1988), as is the case in this work. The regression task begins with the predictor variable set ***X*** (containing pre-to-post-treatment changes in FOCM/TS measurements) and the response variable set ***Y*** (containing pre-to-post-treatment changes in the VABS Composite). To initiate the PLS algorithm, a projection vector for the *n* samples contained in ***X*** is determined and a separate projection vector for the *n* samples contained in ***Y*** is identified. The projections of ***X*** and ***Y*** are then used to calculate the regression coefficients for the model. Further projection directions can be found by subtracting the contributions of the previous directions from ***X*** and ***Y*** and repeating the regression procedure.

Kernel partial least square regression first carries out a non-linear transformation of the form ***F*** = Φ(***X***) on the predictor set, with the dimension of ***F*** typically much larger than that of ***X***. The algorithm then proceeds in a modified form of linear PLS to identify the regression model for predicting ***Y*** from ***F***, rather than from ***X***. Gaussian kernel functions are used in this work for the non-linear transformation Φ(***X***). Here, ***X*** contains the pre-to-post-treatment changes of a subset of the measured metabolites and ***Y*** describes the pre-to-post-treatment change in the VABS Composite. Figure 1B provides a summary of these predictor and response variables used to develop the regression model.

#### Cross-Validation

Classification and regression analyses presented in this study make use of leave-one-out cross-validation to provide a statistically independent assessment of model predictions. This technique removes one sample from the data set, identifies the FDA or KPLS model that fits the remaining data, and then uses the model to predict the sample that was removed. The sample is then replaced and the procedure repeated until all samples have been individually removed once. For classification, the confusion matrix is then constructed using the cross-validated predictions instead of the fitted discriminant scores. Similarly, the sum of squared errors for assessing a regression model is computed as the difference between the measured and the predicted, rather than the fitted, values. Approaching the modeling tasks in this manner helps to alleviate concerns of over-fitting that may arise during model development.

## Results

### FDA Model Identification From IMAGE Data

**FIGURE 2:**
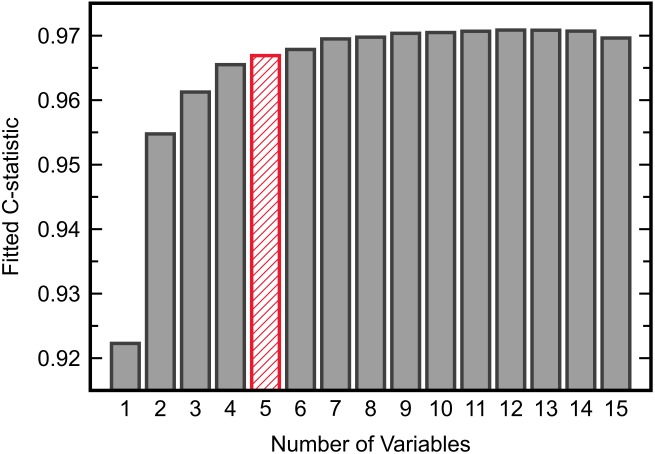
The maximum C-statistic from FDA model fitting across all possible combinations of each given number of input variables. Five variables provided adequate separation between the ASD and TD cohorts in the IMAGE data set.

Development of an FDA model for ASD/TD classification first required the identification of an optimal set of variables to be included in the model. Inclusion of all 15 available measurements was undesirable as overfitting on the training data then becomes more likely due to a low observation-to-variable ratio; this subsequently increases the likelihood of prediction error when validating the model on new data. It was thus of initial interest to determine the smallest set of variables that yielded a satisfactory separation between the ASD and TD cohorts described in the IMAGE data set. To this end, all possible combinations of measurements were exhaustively tested with FDA. The separation between cohorts offered by each variable combination was quantified by the C-statistic resulting from model fitting; the C-statistic is one metric for assessing classifier performance and its value ranges from 0.5 (random guessing) to 1.0 (perfect classification). Upon reviewing the maximum C-statistic obtained for each given number of variables (Figure 2), it was determined that five variables, giving a C-statistic of 0.967, offered a balance between low numbers of variables and strong classifier performance.

**FIGURE 3:**
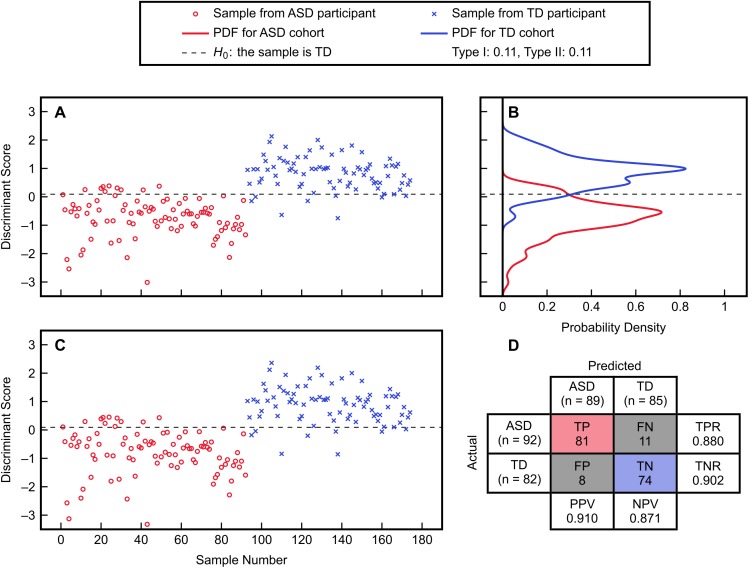
FDA fitting and cross-validation results using the variables methionine, cysteine, Cys-Gly, GSSG, and percent oxidized from the IMAGE data set. **(A)** Discriminant scores from the fitted FDA model for the ASD and TD cohorts. **(B)** Estimated PDFs of the fitted discriminant scores for each cohort, with associated Type I and Type II errors of 11% each. **(C)** Discriminant scores predicted from leave-one-out cross-validation. **(D)** Confusion matrix detailing the true positive (TP), true negative (TN), false positive (FP), and false negative (FN) predictions from cross-validation. The true positive rate is TPR = TP/(TP + FN), the true negative rate is TNR = TN/(TN + FP), the positive predictive value is PPV = TP/(TP + FP), and the negative predictive value is NPV = TN/(TN + FN).

**FIGURE 4:**
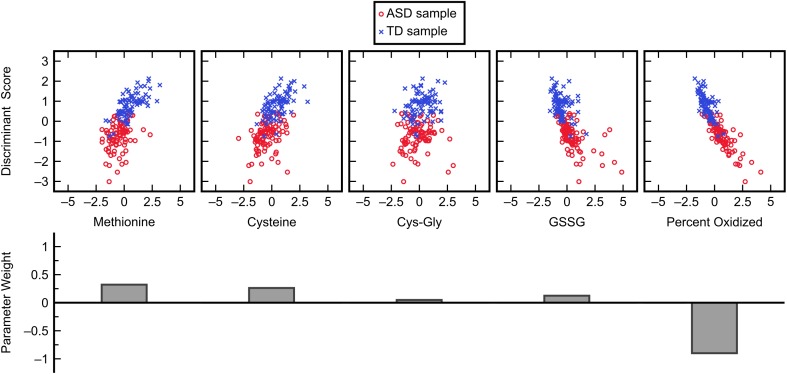
Influence of each normalized input variable in the FDA model. Glutathione precursors and redox measurements cumulatively dominate the ASD/TD classification decision in this model with some less significant contributions by methylation metabolites.

The subset of five variables including methionine, cysteine, Cys-Gly, GSSG, and percent oxidized was selected as the best candidate and was then evaluated with FDA using cross-validation (Figure 3). This model predicted the left-out samples with a sensitivity of 88.0% and specificity of 90.2%, indicating very good classification accuracy. Investigation of each variable’s contribution to the model’s discriminant score (Figure 4) revealed that individuals’ scores were most strongly associated with their measurement for percent oxidized glutathione; study participants classified as TD typically had lower percentages of oxidized glutathione compared to those classified as having ASD. The variable with the second greatest influence in the model was methionine, which was the only included methylation metabolite among a large majority of glutathione precursors and redox measurements (cysteine, Cys-Gly, GSSG, and percent oxidized).

**FIGURE 5:**
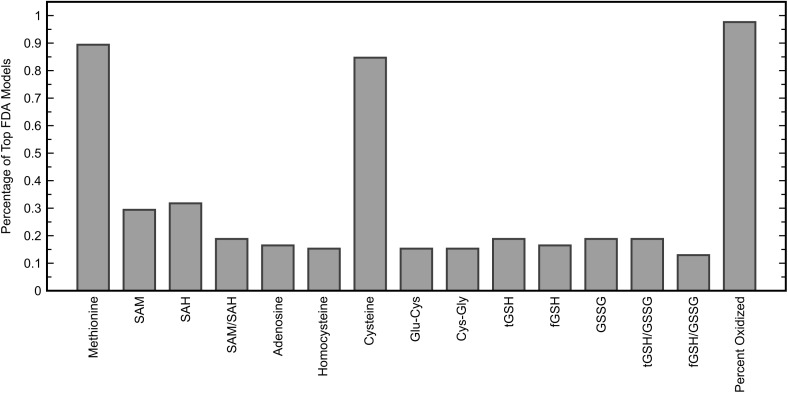
Percentage of five-variable FDA models that each input variable appeared in, among models yielding a fitted C-statistic of 0.96 or greater (85 models). The five variables used in the FDA model with the highest C-statistic were methionine, cysteine, Cys-Gly, GSSG, and percent oxidized.

While the chosen FDA model included many more glutathione precursors and redox markers than methylation metabolites, it was important to determine if this held up for other models that performed almost as well. To investigate individual variable contributions beyond the best model, the frequencies with which each of the fifteen measurements were used in five-variable models offering a fitted C-statistic of 0.96 or greater were considered (Figure 5). This criterion was satisfied by 85 FDA models, out of a possible 3003 five-variable models overall, and it was found that the variables methionine, cysteine, and percent oxidized each appeared in more than 84% of these models while no other measurement was used in more than 32% of models. The measurement of percent oxidized, specifically, was used in almost 98% of the top models, reinforcing its importance for distinguishing participants in the ASD and TD cohorts.

### Treatment Effects on Overall Metabolic Status

**FIGURE 6:**
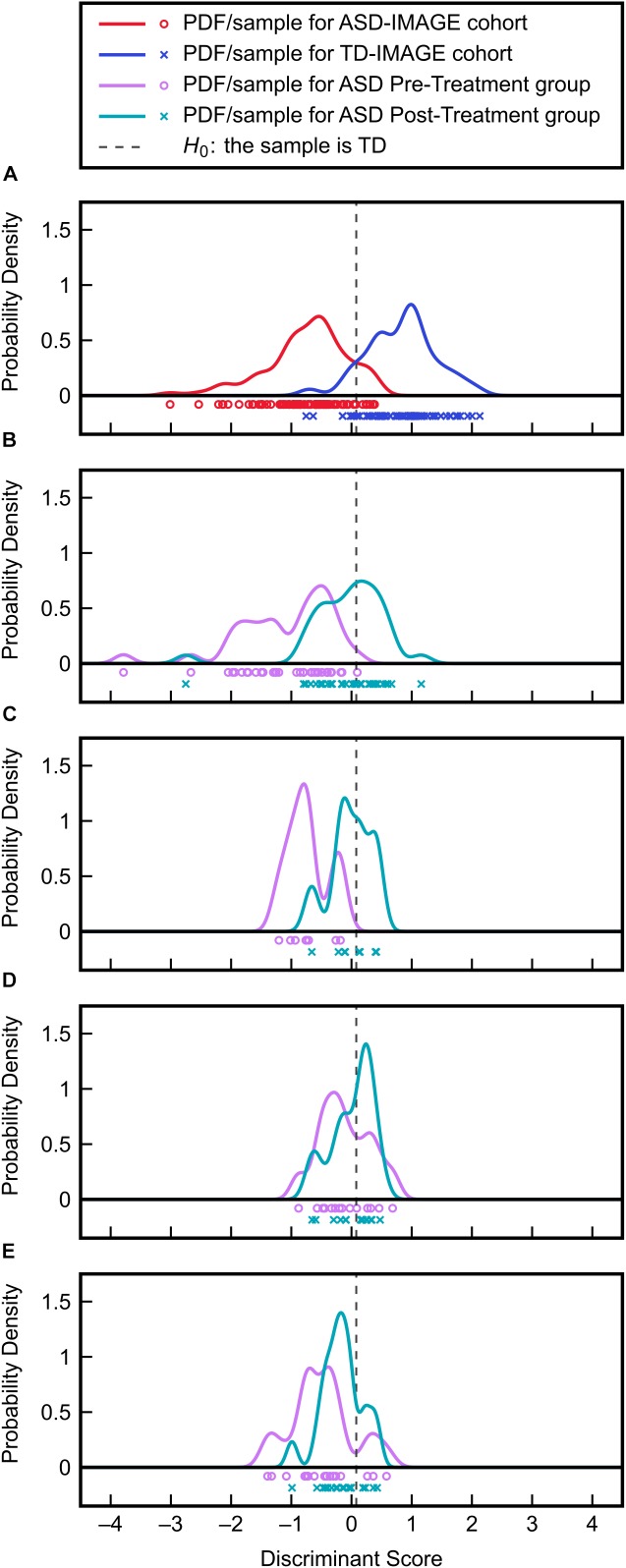
Validation of the FDA model on clinical treatment data. **(A)** Discriminant scores and PDFs for the ASD and TD cohorts from the IMAGE study provide the reference distributions for evaluating the effects of treatment. **(B)** Discriminant score distributions before and after MeCbl + LDFA treatment. **(C)** Discriminant score distributions before and after BH_4_ treatment. **(D)** Discriminant score distributions before and after HDFA treatment. **(E)** Discriminant score distributions before and after placebo treatment.

To assess the efficacies of each clinical treatment (MeCbl + LDFA, BH_4_, HDFA, and placebo) to correct metabolic abnormalities in individuals with ASD, pre- and post-treatment observations from the four groups were evaluated with the identified FDA model. PDFs of the resulting discriminant scores were estimated and compared to the ASD and TD distributions generated from the IMAGE data (Figure 6); the treatments producing the largest pre-to-post-treatment shifts toward the TD distribution can be understood as those offering the greatest improvements to overall FOCM/TS metabolic status. These shifts were quantified as the change in Type II error associated with the PDFs, with respect to the null hypothesis *H*_0_, brought about by each treatment. MeCbl + LDFA produced the largest increase in Type II error (Table 2), followed by BH_4_, indicating that these treatments were the most successful in altering the FOCM/TS profiles of individuals with ASD to more closely reflect those of TD individuals. Treatment with HDFA produced a relatively small increase in Type II error; however, due to the Type II error in this group being very large initially, the post-HDFA treatment error was actually the greatest among all treatments. As a result, the 95% CI for the effect size of HDFA contained zero, whereas the 95% CIs for the other treatments did not contain zero. The 95% CI for the placebo unexpectedly did not contain zero, indicating a small, but statistically significant, metabolic shift in this group.

**Table 2:** Changes in type II error associated with the PDFs of discriminant scores before and after each treatment, with respect to the null hypothesis *H*_0_.

Treatment	Pre-treatment Type II error (%)	Post-treatment Type II error (%)	Change in Type II error (%)	Effect size (95% CI)
MeCbl + LDFA	1.7	43.6	+41.9	0.89 (0.68, 1.40)
BH_4_	0.3	41.1	+40.8	0.73 (0.31, 1.11)
HDFA	32.2	49.5	+17.2	0.17 (-0.21, 0.46)
Placebo	15.1	21.3	+6.20	0.31 (0.12, 0.60)


*Effect size was calculated as the median change in pre-to-post-treatment discriminant score, where pre-treatment samples were paired with their post-treatment data points.*

### Prediction of Changes in Adaptive Behavior

It has been established that treatments with MeCbl + LDFA, BH_4_, and HDFA offer varying levels of improvement in metabolic status in individuals with ASD. The next part of this investigation involved the use of KPLS regression to predict changes in adaptive behavior (as quantified by the VABS Composite score) from changes in FOCM/TS measurements resulting from treatment. Thus, the predictor variables in the regression were the pre-to-post-treatment changes in metabolic measurements while the response variable was the pre-to-post-treatment change in VABS Composite. All treatment groups, including the placebo group, were included in the regression (74 samples in total) so as to capture a range of biochemical/behavioral effects and to further guard against overfitting by using metabolites from as many participants as possible. It should also be noted that this analysis is independent of the treatment used as only the changes in the pre-to-post-treatment are correlated with changes in the VABS scores and information about the treatment itself is not used for regression. That being said, the type of treatment used affects the pre-to-post-treatment changes in the metabolites, but the treatment information is only implicitly and not explicitly involved in this analysis.

**FIGURE 7:**
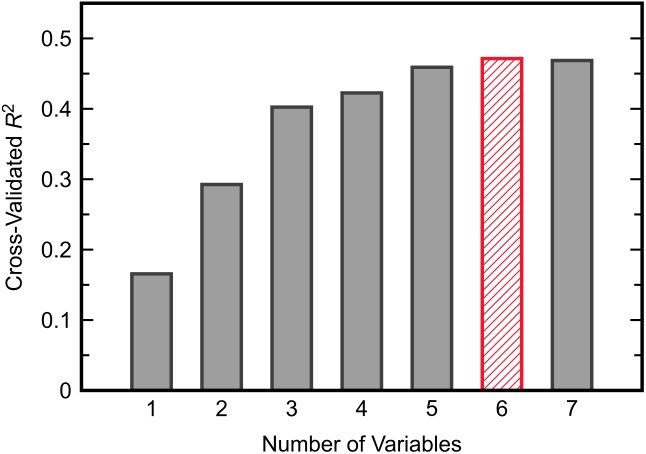
The maximum value of *R*^2^ from cross-validated KPLS regression across all possible combinations of each given number of predictor variables.

**FIGURE 8:**
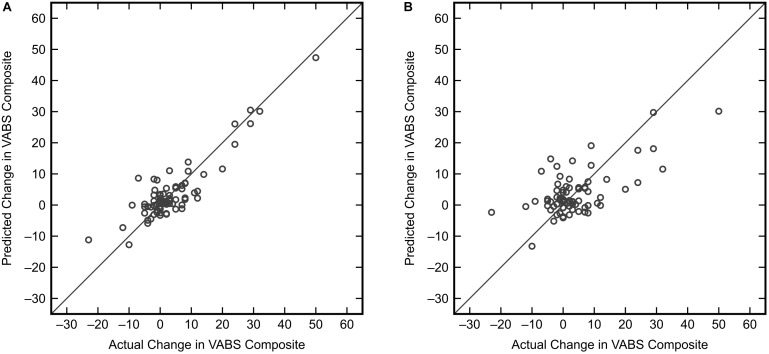
KPLS regression model predictions versus actual changes for the VABS Composite score using the best combination of six predictor variables (Δmethionine, ΔGlu-Cys, ΔCys-Gly, ΔtGSH, ΔtGSH/GSSG, ΔfGSH/GSSG). **(A)** Predictions from the fitted model (*R*^2^ = 0.796). **(B)** Predictions resulting from model cross-validation (*R*^2^ = 0.471).

**FIGURE 9:**
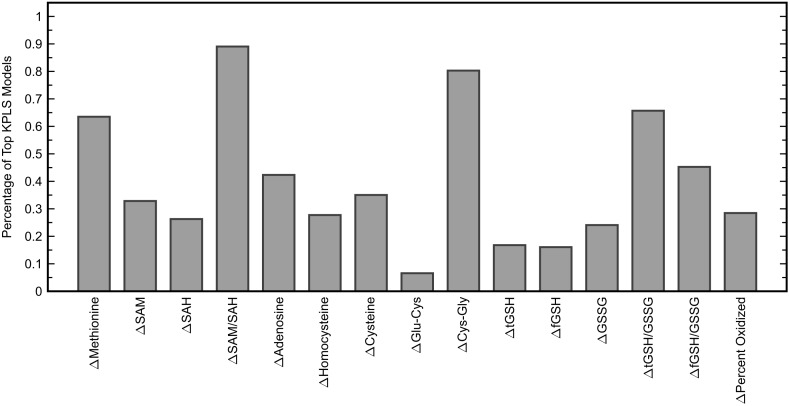
Percentage of six-variable KPLS regression models that each predictor variable appeared in, among models yielding a cross-validated *R*^2^ of 0.35 or greater (137 models). The six variables used in the KPLS model with the highest cross-validated *R*^2^ were Δmethionine, ΔGlu-Cys, ΔCys-Gly, ΔtGSH, ΔtGSH/GSSG, and ΔfGSH/GSSG.

Similar to the classification task, the initial step for this analysis was to identify an appropriate subset of input variables for the KPLS model. All combinations of each number of variables were exhaustively tested and the *R*^2^ from cross-validation was used as the evaluation criterion for the regression. Comparing the maximum *R*^2^ given by each number of input variables (Figure 7) showed the model performance to decrease when more than six variables were used. The highest cross-validated *R*^2^ of 0.471 was obtained using Δmethionine, ΔGlu-Cys, ΔCys-Gly, ΔtGSH, ΔtGSH/GSSG, and ΔfGSH/GSSG as predictor variables in the regression, where Δ indicates the pre-to-post-treatment change of a particular metabolite or metabolite ratio. The predictions from fitting and cross-validation with this model are provided in Figure 8. Although this particular combination of variables gave the best model performance under cross-validation, a number of other combinations offered similar prediction accuracy; the five top-performing models using six predictor variables are listed in Table 3 to demonstrate this point. The frequency of appearance for all fifteen study variables in these combinations was considered across all six-variable models producing an *R*^2^ of 0.35 or greater after cross-validation (Figure 9); in total, 137 combinations (out of a possible 5005) met this criterion. Methylation metabolites, glutathione precursors, and redox markers appeared with overall similar frequency in these top combinations, although the single model with the highest cross-validated *R*^2^ was dominated by glutathione precursors and redox measures.

**Table 3:** The five combinations of predictor variables producing the highest *R*^2^ from cross-validation with KPLS regression when using six variables.

Variables	*R*^2^
ΔMethionine, ΔGlu-Cys, ΔCys-Gly, ΔtGSH, ΔtGSH/GSSG, ΔfGSH/GSSG	0.471
ΔMethionine, ΔSAM/SAH, Δadenosine, Δcysteine, ΔCys-Gly, tGSH/GSSG	0.470
ΔMethionine, ΔSAM, ΔSAM/SAH, Δadenosine, Δcysteine, ΔCys-Gly	0.467
ΔSAM, ΔSAM/SAH, Δhomocysteine, Δcysteine, ΔCys-Gly, ΔtGSH/GSSG	0.462
ΔMethionine, ΔSAM/SAH, Δadenosine, ΔCys-Gly, ΔtGSH/GSSG, ΔfGSH/GSSG	0.454


## Discussion

Classification of ASD and TD cohorts in this work showed that multivariate analysis can uncover very good separation between these groups with a classifier sensitivity of 88.0% and specificity of 90.2%. Previous work by the authors used FDA on the same data from the IMAGE study to achieve correct classification in 97.6% of individuals with ASD and 96.1% of TD individuals (Howsmon et al., 2017). That study had the advantage of using additional measures of DNA methylation (percent methylated DNA), chronic oxidative stress (8-hydroxyguanosine, chlorotyrosine, and nitrotyrosine), redox metabolism (free cystine, free cysteine, and free cystine/free cysteine), and amino acids (tyrosine, tryptophan) for classification that were not available for the current work due to our use of additional data sets where some or all of these measurements were not available. As a result of using fewer measurements, however, we were able to include additional samples from the IMAGE data set that were previously omitted on the basis of them missing one or more of the aforementioned measurements; this provided 92 ASD and 82 TD in the current study compared to the 83 ASD and 76 TD reported previously (Howsmon et al., 2017). Thus, the discrepancy in classification accuracy may arise from the larger group of samples, but differences in measurement availability likely have a larger role.

Our identified FDA model placed significant weight on FOCM/TS measurements related to oxidative stress while measurements linked to DNA methylation were of less importance. The measurement for percent oxidized glutathione especially dominated the classification decision, with the magnitude of its parameter weight greater than the total combined weight given to the other model inputs. This may indicate that processes closely related to glutathione metabolism are more abnormal in individuals with ASD than are DNA methylation processes; it can at least be said that these particular measurements of redox metabolism are more accurate indicators of ASD status than the methylation markers that were considered in this study. Li et al. (2018) did find that the measure of percent DNA methylation has even greater importance to the ASD/TD classification than percent oxidized glutathione, but due to the aforementioned restrictions on our data sets we were unable to include percent DNA methylation in our analyses.

Through evaluation of the FDA model on the clinical trial data sets, the MeCbl + LDFA treatment was found to provide the greatest correction in ASD-related metabolic abnormalities, with the effects of BH_4_ just slightly smaller; both of these treatments increased the rate of ASD misclassification by more than 40% each. It is worth noting that individuals in both of these studies had poor FOCM/TS metabolic status at baseline (James et al., 2009; Frye et al., 2013a), a point that is reflected in the low pre-treatment Type II errors associated with these groups in our analysis. These groups were thus expected to be the most responsive to treatment and the large shift in PDFs resulting from these treatments could be partly explained by these baseline characteristics. In contrast, the Type II error was large in the HDFA group even before treatment, leaving less room for improvement with the treatment. The post-HDFA treatment participants did have nearly a 50% misclassification rate, indicating some ability of HDFA to improve metabolic status despite its 95% CI for effect size containing zero. However, given that the initial measurements in the HDFA group were considerably better than those for the MeCbl + LDFA and BH_4_ groups, it is difficult to state whether HDFA could have offered a more significant metabolic correction than what was observed. It should be mentioned that the HDFA treatment was not aimed at correcting peripheral metabolism, but was rather designed to increase deficient central folate metabolism. These data in fact support the mechanism of action proposed for the HDFA, which is to increase central folate, rather than change peripheral metabolism since the behavioral effect of HDFA was strong despite little change in peripheral metabolism. This also supports the notion that HDFA can complement these other treatments in order to improve multiple pathophysiological abnormalities associated with ASD. Finally, the placebo group expectedly had a relatively small change in Type II error, but the effect size from placebo treatment was unexpectedly significant. This could possibly be attributed to a decrease in the percent oxidized glutathione that was measured in a number of participants receiving the placebo, which would explain the shift of the PDF toward the TD side.

All treatments clearly provided some degree of metabolic correction. Despite the improvements, however, no treatment came close to offering complete normalization of FOCM/TS metabolism; the highest ASD misclassification rate was 49.5% in the HDFA treatment group. Based on the results of the initial FDA model identification, total normalization of a treatment group would be achieved when 11% of the distribution is classified as ASD, or similarly when the Type II error is 89%. There is thus a significant gap between the observed effect and what could potentially be achieved, and while this is not unexpected given the complex pathophysiology of ASD, it does give an indication of the magnitude of metabolic abnormalities even after treatment.

Our finding that changes in adaptive behavior could be regressed onto changes in biochemical measurements with an *R*^2^ of 0.471 after cross-validation indicates that deviations from a person’s baseline FOCM/TS measurements correlate significantly with deviations from their baseline behavior. This may be suggestive of a biological mechanism by which abnormalities in FOCM/TS processes lead to certain alterations in neurological activity; it is also possible that the converse is true. Importantly, a number of variable combinations produced very good *R*^2^ values after cross-validation, with several metabolites appearing in many top-performing combinations even though no measurements appeared in all combinations and even the least predictive measurements appeared in some combinations. This can be explained by the interconnected structure of FOCM and TS where the effects of metabolites are non-linearly dependent on one another (Vargason et al., 2017a); for example, some biochemical measurements may be predictive of behavioral changes only when they are grouped with a specific subset of other biochemical measurements, and less informative when considered independently of these other measures. The lesser importance of glutathione/redox measurements for prediction of adaptive behavior relative to the importance of these measurements in the classification task may be explained by KPLS uncovering certain non-linear relationships that the linear analysis of FDA did not describe; it is also possible that the glutathione/redox measurements simply correlate more strongly with ASD or TD status than they do with adaptive behavior. Finally, the significance of our result arising from cross-validation must be emphasized, as it indicates that the regression model is capable of predicting new data not used during model training, a conclusion that could not be made from a fitted result alone (Frye et al., 2013b).

It could be argued that the placebo group should not be included in the regression analysis since significant changes in metabolites or behavior should not be observed in this group. However, it is possible that small fluctuations in metabolic activity may still correlate to small changes in adaptive behavior regardless of whether these changes are due to a treatment or are happening because of other factors. Ideally, a model would be able to capture changes both large and small arising from these sources, and while the treatment groups provide information about the former, the latter would be uncharacterized without analyzing the placebo group. The inclusion of participants receiving the placebo also substantially increases the number of samples for model training and cross-validation, providing further safeguard against overfitting. Considering these perspectives, using the placebo group is likely to give a more robust estimate of the correlation between metabolic and behavioral changes.

Interpretation of the presented results may be influenced by limitations in the design of the current study as well as those of the studies from which the clinical data were obtained. The clinical limitations have been previously discussed in their respective studies (James et al., 2009; Melnyk et al., 2012; Frye et al., 2013a, 2016b), so only the limitations directly relevant to the current study’s design will be discussed here. One of the largest sources of error in this study arises from its use of data from four separate clinical data sets, which may introduce variation into the biochemical and behavioral measurements with regard to the procedures and study populations used for data collection. Different baseline characteristics for the separate treatment groups affect our ability to directly compare the effects of each treatment, since the magnitudes of these effects likely depend on the initial severity of the metabolic abnormalities, that is, someone with very irregular metabolic status may have much more potential for improvement than a person with less severe abnormalities. Another important limitation is the small samples sizes used in some of the studies, which restricted our ability to find statistically meaningful differences. As a direct result of this, many of our findings are suggestive of substantial treatment effects, but not as significant as would be desired from these treatments. With the BH_4_ data in particular, the inclusion of only eight participants could make the evaluation of meaningful effects more challenging; however, since these data alone were not used to fit models and were used only for model validation (as with FDA) or when combined with other data sets (as with KPLS), this concern is at least partially indirectly addressed. Averaging the 8- and 16-week measurements from the BH_4_ study to obtain a 12-week estimate also has some associated risk as certain measurements might not scale linearly between the two time points. Additionally, VABS Composite scores reported in the open-label trials are susceptible to expectation bias and it cannot be said with certainty if these reported scores accurately reflect the true adaptive behavior observed at the time of the trial. A final point of note is that variations in cognitive levels of participants could potentially have effects on treatment outcomes, but we were unable to assess these effects since intelligence measures were not available in the clinical data.

Everything considered, the multivariate analysis performed in this study offers further insight into the metabolic and behavioral improvements resulting from clinical treatment of individuals with ASD. Classification via FDA model identification and validation provided additional measures of treatment efficacy that went beyond univariate comparisons of individual measurements, and instead considered the combined contributions of multiple markers toward FOCM/TS metabolic status. The use of KPLS regression to predict changes in adaptive behavior from changes in metabolites also represents an important step toward the development of a clinical trial target for ASD that captures both metabolic and behavioral effects offered by a particular treatment. Together, these results suggest that dysfunction in metabolism and the brain in individuals with ASD may not occur as isolated systems, but rather as one connected system in ASD pathophysiology.

## Data Availability Statement

The raw data supporting the conclusions of this manuscript will be made available by the authors, without undue reservation, to any qualified researcher.

## Author Contributions

TV, UK, and JH contributed to the conception and design of the current study. TV, UK, and ER performed the data analysis presented in the current study. LD, MT, and JS collected the clinical data and samples. SR, SB, and SM processed the clinical samples and performed the assays. SJ and RF designed the clinical studies. TV wrote the first draft of the manuscript. TV, UK, JS, RF, and JH contributed to revision of the manuscript. All authors read and approved of the submitted manuscript.

## Conflict of Interest Statement

JS and SJ are founding members of BioROSA Technologies, Inc., which seeks to develop diagnostic tests based upon FOCM/TS metabolites. However, their contribution to this work concluded before BioROSA Technologies, Inc. was founded. The remaining authors declare that the research was conducted in the absence of any commercial or financial relationships that could be construed as a potential conflict of interest.
